# HPLC-DAD analysis, antioxidant potential and anti-urease activity of *Asparagus gracilis* collected from District Islamabad

**DOI:** 10.1186/1472-6882-14-347

**Published:** 2014-09-23

**Authors:** Naseer Ali Shah, Muhammad Rashid Khan, Saadia Sattar, Bushra Ahmad, Bushra Mirza

**Affiliations:** Department of Biochemistry, Faculty of Biological Sciences, Quaid-i-Azam University, Islamabad, 45320 Pakistan; Department of Biosciences, COMSATS Institute of Information Technology, Islamabad, Pakistan

**Keywords:** Antiurease, Phytochemistry, Antioxidant, Mutltipotent activity, Fractionation

## Abstract

**Background:**

*Asparagus gracilis* subspecie of Asparagus capitatus Baker, is described as food and medicine for various ailments. In this study we investigated, its phenolic constituents, *in vitro* antioxidant potential against various free radicals and anti-urease potential.

**Methods:**

*Asparagus gracilis* aerial parts collected from District Islamabad, Pakistan were extracted with crude methanol which was further fractionated into *n*-hexane, ethyl acetate, *n*-butanol and aqueous fraction. Total phenolic and flavonoid contents were estimated for extract and all the derived fractions. Diverse *in vitro* antioxidants assays such as DPPH, H_2_O_2_, •OH, ABTS, β-carotene bleaching assay, superoxide radical, lipid peroxidation, reducing power, and total antioxidant capacity were studied to assess scavenging potential. Antiurease activity of methanol extract and its derived fractions was also investigated. HPLC-DAD analysis of crude methanol extract was performed by using different phenolic standards.

**Results:**

Ethyl acetate fraction expressed maximum content of flavonoids (240.6 ± 6.1 mg RE/g dry sample), phenolics (615 ± 13 mg GAE/g dry sample) and best antioxidant potential among different fractions of crude methanol extract. Hydrogen peroxide assay and hydroxyl, supeoxide, nitric oxide free radicals antioxidant assays as well as beta carotene assay showed significant correlation with flavonoid content while hydrogen peroxide, ABTS and lipid peroxidation assay displayed significant correlation with phenolic content. HPLC analysis showed the presence of important phenolics i.e. catechin (4.04 ± 0.02 μg/mg sample), caffeic acid (0.89 ± 0.003 μg/mg sample), rutin (24.58 ± 0.1 μg/mg sample), myricetin (1.13 ± 0.07 μg/mg sample) and quercetin (14.91 ± 0.09 μg/mg sample). Ethyl acetate fraction expressed lowest IC_50_ in antiurease activity. Correlation analysis of antiurease activity expressed significant correlation with flavonoids (P < 0.004) and phenolics (P < 0.02) proposing multipotent activity of fractions.

**Conclusion:**

These results revealed the presence of some bioactive compound in the ethyl acetate fraction having both antioxidant as well as antiurease potential.

## Background

Free radicals such as reactive oxygen species (ROS) and reactive nitrogen species (RNS) are classified having (or not) one or more unpaired electron. They belong to very reactive species, produced continuously in cells as normal metabolic products. They perform both beneficial as well as harmful roles in living body. ROS play important role in physiological mechanism such as induction of mitogenic responses, cellular signaling pathways and responses to infectious agents at low and moderate concentrations while RNS play important role in blood pressure maintenance, neurotransmission, defense mechanism, immune regulation and smooth muscle relaxation. In biological systems, overproduction of ROS and RNS results in state termed oxidative stress and nitrosative stress, respectively. These condition occur, when there is overproduction of ROS/RNS and enzymatic or non enzymatic antioxidants are not satisfying the required level. If this state persists for a longer period, it may lead to many clinical disorders such as inflammatory diseases, aging, asthma, diabetes mellitus, cardiovascular diseases, rheumatoid arthritis and cancer [1].

Oxidation is also playing a deleterious role in the food industry by leading to rancidity of foods rich in unsaturated fatty acids and other compounds having the danger of oxidative degradation [2]. Nowadays, the market is full of synthetically manufactured neutralizing agents having associated problems like toxicity and/or mutation induction, resulting in attention of many researchers to look for natural antioxidant [3]. For natural antioxidants, plants have gained much attention for producing compounds with antioxidant property, showing protection against oxidative stress in humans. In plants, secondary metabolites such as flavonoids are a well known class of antioxidants [4] due to their high redox value, making them as good hydrogen donor, reducing agent and singlet oxygen scavenger [5]. Flavonoids are recognized for their character to hinder oxidative damage of unsaturated fatty acids with rapid and simple metabolic degradation pathways, making them an ideal preservative alternative to synthetic antioxidants such as butylated hydroxytoulene or butylated hydroxyl anisole in food industry [2].

Urease is a prominent component of *Helicobacter pylori*, the causative agent of gastro duodenal diseases resulting in peptic and gastric cancer. In stomach urease convert urea into ammonia which minimizes stomach acidity. Stomach with low acidity provide ideal growth condition for *H. pylori* pathogen and support its colonization. Urease also acts directly as virulence factor in infections of gastrointestinal as well as urinary tract in humans and animals. *H. pylori* is susceptible to antibiotics but treatment failure occurs in more than 15 percent of patients. Natural products are suitable alternate choice for screening of urease inhibition to combat *H. pylori* infection [6–8].

The screening studies for antioxidant properties of medicinal plants and foods have been performed increasingly for the last few decades in hopes of finding an efficient remedy for several present day free radical problems [7] and multipotent active antioxidant compounds [2].

*Asparagus gracilis* is subspecie of *Asparagus capitatus* Baker, locally it is known as Lachghawa in district Dera Ismail Khan (DI Khan), Pakistan. Its young shoots are used as aphrodisiac and diurectic. It is also used as vegetable cooking alone or mixing with eggs [9]. Shah et al. [10] reported its antileishmanial activity. At present, there is no single study regarding its phytochemical constituents and antioxidant activity potential. Therefore, this study was undertaken on the basis of random screening approach to evaluate its chemical constituents, and multi-activity potential by evaluating antioxidant activities and urease inhibition.

## Methods

### Chemicals

All the chemicals used in these assays were of high polarity (99%). Ascorbic acid, gallic acid, rutin, Folin-Ciocalteu’s phenol reagent, AlCl_3_.6H_2_O, 2,2-Diphenyl-1-Picrylhydrazyl (DPPH), 2,2-azino-bis(3-ethylbanzthiazoline-6-sulphonic acid (ABTS), potassium oxidopersulphate, ammonium molybdate, phenazine methosulphate (PMS), nitroblue tetrazolium (NBT), ferric chloride, potassium chloride, trichloroacetic acid (TCA), thiobarbituric acid (TBA), potassium ferricynide, Mayer’s reagent, FeCl_3_ were purchased from Sigma Co. (St. Louis,MO, USA). H_2_SO_4_, 2-deoxyribose riboflavin, Na_2_CO_3_, NaOH, NaNO_2_, H_2_O were purchased from Wako Co. (Osaka, Japan). All analytical grade solvents e.g. *n*-hexane, chloroform, ethyl acetate and *n*-butanol were used with 99.8% purity level and were obtained from Merck Co.(Darmstadt, Germany). Ultrapure TM water purification system (Lotum Co., Ltd., Taipei,Taiwan) was used to get deionized distilled water.

### Plant collection and extract preparation

The plant was collected in March 2012 from the surrounding of the Quaid-i-Azam University, Islamabad, Pakistan. Plant sample was identified by Dr. Mir Ajab Khan, Department of Plant Sciences, Quaid-i-Azam University, Islamabad. A voucher specimen was deposited in the Herbarium of Department of Plant Sciences, Quaid-i-Azam University, Islamabad. Plant material was washed with fresh water to remove dirty material. The dried material was then ground into coarse powder by grinding machine.

Dry powder (10 kg) of *A. gracilis* was socked in 20 liters of crude methanol and shaked a number of times. After 72 hours of soaking, filtered through filter paper (Whatmann filter 1), and the filtrate was concentrated through the rotary vacuum evaporator at reduced pressure to get the crude methanol extract. To sort the crude methanol extract (AGME) in increasing order of polarity it was dissolved in distilled water (6 g/250 ml) and passed through different solvents (250 ml each) in the order of *n*-hexane → ethyl acetate → *n*-butanol to get *n*-hexane fraction (AGHE), ethyl acetate fraction (AGEE) and *n*-butanol fraction (AGBE) by using 500 ml separating funnel. The residue remaining at the end was termed aqueous fraction (AGAE). All the fractions were stored at 4°C until further use.

### Phytochemical analysis

#### Total phenolic content estimation

Spectrophotometric method [11] was used for determination of total phenolic content. In short, 1 ml of the extract and its derived fractions (1 mg/ml) were mixed with 1 ml of Folin-Ciocalteu’s reagent. To the mixture, 10 ml of 7% Na_2_CO_3_ was added after 5 min, followed by 13 ml of deionized distilled water and allowed to mix thoroughly. The mixture was incubated at 23°C in the dark for 90 min. Absorbance was recorded at 750 nm. Total phenolic content was calculated from calibration curve of gallic acid. Estimation of TPC was recorded in triplicate and was presented as mg of gallic acid equivalents (GAE) per g of dry sample.

#### Total flavonoid content estimation

In a test tube, 0.3 ml of the sample, 3.4 ml 30% methanol, 0.15 ml of 0.5 M NaNO_2_ and 0.1 ml of 0.3 M AlCl_3_.6H_2_O were thoroughly mixed in a test tube. After 5 min, 1 ml of 1 M NaOH was added and mixed well. Absorbance was measured at 506 nm against the reagent blank. Total flavonoid content was estimated by using a calibration curve of rutin and expressed as mg rutin equivalents per g of dry sample [11].

### HPLC analysis

Methanol, acetonitrile and acetic acid were of HPLC grade (Tedia Company, USA) while deionized water was prepared by a Milli-Q Water Purification system (Millipore, MA, USA). Nine reference standards were used, i.e. Cathechin, rutin, kaemferol, quercetin, gallic acid, salicylic acid, apigenin, myricetin and caffeic acid (Sigma company, USA). Standards and plant plant sample solutions were prepared in methanol, at a concentration of 100 and 10 mg/ml respectively. Samples were filtered through 0.45 μm membrane filter.

Chromatographic analysis was carried out by using HPLC-DAD attached with C-18 (Discovery) analytical column. Briefly, mobile phase A was acetonitrile-methanol–water-acetic acid (5:10:85:1) and mobile phase B was acetonitrile- methanol- acetic acid (40:60:1). A gradient of time 0–20 minutes for 0 to 50% B, 20–25 minutes for 50 to 100% B and then isocratic 100% B till 30 minutes was used. The flow rate was 1 ml/min and injection volume was 20 μl. All the samples were analyzed at 257 nm wavelength. Each time column was reconditioned for 10 minutes before the next analysis. All samples were assayed in triplicate. Quantification was carried out by the integration of the peak using the external standard method. All chromatographic operations were carried out at ambient temperature.

### Antioxidant capacity determination assays

An amount of 200 μg of plant samples and positive standards (Ascorbic acid, Butylated hydroxytoluene, Catechin and Gallic acid) was prepared in one ml analytical methanol. These solutions were further diluted to 100, 50, 25, 12.5 μg/ml. In all the different antioxidant assays, same dilutions of sample and standards were used; while standard alter per assay requirement.

#### DPPH radical scavenging assay

The DPPH (1, 1-diphenyl-2-picryl-hydrazyl) bioassay was performed according to the slight modification of protocol of Said et al. [12]. Working solution of DPPH with absorbance of 0.98 ± 0.02 was obtained at 517 nm from the standard solution of DPPH (0.24 mg/ml). A volume of 0.9 ml of DPPH solution was mixed with 100 μl of various concentrations of test samples and incubated for 60 min in the dark at room temperature. Absorbance was recorded at 517 nm. Scavenging activity was calculated using the following equation;


#### Hydrogen peroxide scavenging assay

The method of Ahmad et al. [13] was followed to investigate hydrogen peroxide scavenging capacity of samples. A solution of 2 mM hydrogen peroxide was prepared in 50 mM phosphate buffer (pH 7.4). A volume of 100 μl of the sampls was added in 300 μl of 50 mM phosphate buffer (pH 7.4). Absorbance of the mixture was recorded at 230 nm after 10 min of incubation by following the addition of 600 μl of hydrogen peroxide solution. Percent scavenging activity was determined by following formula;


Ascorbic acid served as standard.

#### Hydroxyl radical scavenging assay

Hydroxyl radical scavenging activity was evaluated by the reported method of Sahreen et al. [14]. The reaction mixture was prepared by the addition of 500 μl of 2.8 mM 2-deoxyribose in 2 mM phosphate buffer (pH 7.4), 100 μl of 200 mM hydrogen peroxide solution, 200 μl of 100 mM ferric chloride and 100 μl of the test sample. The reaction was initiated by the addition of 100 μl of 300 mM ascorbate and incubated at 37°C for 60 min. To mixture 1 ml of trichloroacetic acid (2.8%; w/v) and 1 ml of thiobarbituric acid (TBA) solution in 50 mM of sodium hydroxide (1%; w/v) was added. The reaction mixture was incubated for 15 min in a boiling water bath and then allowed to cool to room temperature. Absorbance of the mixture was recorded at 532 nm. Catechin was used as standard.


#### ABTS radical cation scavenging activity

Ahmad et al. [13] methodology with slight modification was followed for ABTS (2, 2 azobis, 3-ethylbenzothiozoline-6-sulphonic acid) radical scavenging activity. ABTS (7 mM) solution was reacted with 2.45 mM potassium persulfate and kept overnight in dark for generation of dark colored ABTS radicals. For the assay, the solution was diluted with 50% ethanol for an initial absorbance of 0.7 at 745 nm. To determine the ABTS radical scavenging activity, 100 μl of sample was mixed with 1 ml of ABTS solution in glass cuvette. Decrease in absorbance was measured after 1 and 6 min of mixing. The difference was calculated and compared with control. Percent inhibition was calculated by following formula;


#### Anti lipid peroxidation assay

This assay was performed as illustrated by Dorman et al. [15]. A mixture of egg yolk (10%, w/v) was prepared in KCl (1.15%, w/v). Egg yolk was homogenized for 30 seconds and subsequently subjected to ultrasonication for 5 min. A volume of 100 μl of sample was added in 500 μl of yolk homogenate and volume was made up to 1 ml with distilled water. 1.5 ml of 20% acetic acid (pH 3.5) and thiobarbituric acid (0.8%, w/v) in sodium dodecyl sμlphate (1.1%, w/v) were thoroughly mixed and incubated for 60 min in a water bath. *n-*Butanol was added after cooling at room temperature, stirred and then centrifuged for 10 min at 3000 rpm. The absorbance of supernatant was recorded at 532 nm. Catechin was used as standard.


Where


#### β-Carotene bleaching assay

Khan et al. [16] modified method was used for β-carotene bleaching assay. β-carotene (2 mg) was dissolved in 10 ml of chloroform and mixed with 20 mg of linoleic acid and 200 mg of Tween 80 followed by chloroform removal under nitrogen. Subsequently, 50 ml of distilled water was mixed and shaked vigorously to prepare β-carotene linoleate emulsion. An aliquot of sample (50 μl) was mixed with 1 ml of the emulsion, and absorbance was determined at 470 nm immediately against the blank solution. Capped tube was then kept in a water bath at 45°C for 2 h and the difference in the initial reading was calculated by measuring the reading after 2 h. β-Carotene bleaching inhibition was estimated as the following equation;


#### Superoxide anion radical scavenging assay

Riboflavin light NBT system assay was followed for superoxide radical scavenging activity [17]. The reaction mixture contained; 0.5 ml of 50 mM phosphate buffer (pH 7.6), 0.3 ml of 50 mM riboflavin, 0.25 ml of 20 mM PMS, and 0.1 ml of 0.5 mM NBT, prior to the addition of 1 ml of each sample. Florescent lamp was used for starting the reaction. Absorbance was recorded at 560 nm after incubation for 20 min under light. The percent inhibition of superoxide anion generation was calculated using the following formula;


#### Nitric oxide radical scavenging activity

Ebrahimzadeh et al. [18] protocol was used for estimation of nitric oxide scavenging activity of *A. gracilis* extracts and its various fractions. This protocol based on the principle that sodium nitroproside at physiological pH in an aqueous solution and aerobic condition generates nitric oxide which further reacts with oxygen to form nitrite ions, which is estimated by using Griess reagent. Scavengers of nitric oxide react with oxygen, resulting in low quantity of nitrite ions. In this assay, 10 mM sodium nitroprusside in phosphate buffered saline was mixed with samples and incubated for 150 min at room temperature. After incubation, Griess reagent (0.5 ml) was added and absorbance was taken at 546 nm by a spectrophotometer. The experiment was repeated in triplicate.

#### Reducing power activity assay

The reducing power of *A. gracilis* samples was determined following modified protocol reported by Saeed et al. [11]. The reaction mixture was prepared by the addition of 100 μl of test samples (12.5, 25, 50, 100 and 200 μg/ml), 100 μl of 200 mM phosphate buffer (pH 6.6) and 100 μl of potassium ferricyanide (10 mg/ml) followed by incubation at 50°C for 30 min. An aliquot of 0.25 ml of 1% trichloroacetic acid was added to the mixture. From the mixture, 0.25 ml was mixed with 0.25 ml distilled water and 0.4 ml ferric chloride (0.1% w/v). Absorbance was recorded at 700 nm after 30 min of incubation at room temperature. Increased absorbance is indicative of high reducing power.

#### Total antioxidant (Phosphomolybdate assay)

The total antioxidant capacity of the samples was investigated by phosphomolybdate [11]. An aliquot of 0.1 ml of each sample was mixed with 1 ml of reagent (0.6 M H_2_SO_4_, 0.028 M sodium phosphate, 0.004 M ammonium molybdate) and incubated for 90 min at 95°C in a water bath. Absorbance was recorded at 765 nm after the mixture cooled to room temperature. Ascorbic acid served as a standard.

#### DNA protection assay

The antioxidant potential of *A. gracilis* samples was evaluated by conducting DNA protection assay [19]. Plasmid DNA (pBR322 Fermentas) 0.5 μg/3 μl was treated with 5 μl of each sample (100, 50 and 25 μg/ml). In the reaction mixture 4 μl of 30% H_2_O_2_ and 3 μl of 2 mM FeSO_4_ was used for Fenton reaction induction. Untreated DNA, treated DNA with 2 mM FeSO_4_, DNA treated with 30% H_2_O_2_, and DNA treated with 2 mM FeSO_4_ and 30% H_2_O_2_ was run simultaneously as a control. The reaction mixture was incubated at 37°C for 60 min. Bromophenol blue (3 μl) as a loading dye was added to each reaction mixture after incubation. Samples were run on 1% agarose gel containing ethidium bromide and TBE buffer, and visualized with Doc-IT. Experiment was performed in the dark to avoid photo excitation of samples.

### Antiurease assay

Methodology of Weatherburn [20] based on phenol hypochlorite was adopted to measure the antiurease activity of extract and different fractions of *A. gracilis*. This method basically detects the ammonia produced by the reaction of urease enzyme with urea. The ammonia produced react with reagents of assay and produces a colored complex which shows the amount of ammonia produced. In a microtiter plate, 20 μl of urease enzyme solution (2 U/well) was added to 5 μl of sample solution. After incubating for 20 minutes at 30°C, 40 μl of 100 mM urea solution was added and once again incubated for 25 minutes. Then 70 μl of alkali reagent (0.1% sodium hypochloride and 0.5% NaOH) and 48 μl of phenol reagent (0.005% sodium nitroprusside and 1% phenol) were added and the optical density was measured up to 50 minutes with a gap of 5 minutes using mictroplate reader. Thiourea was used as a standard and mixture without test sample and urea solution was used as a blank. Percentage inhibition of urease by test samples was calculated by using a formula:


### Statistical analysis

All values are mean of triplicates. One way ANOVA analysis was carried out by using Statistix 8.1 to assess the difference between various groups. The graph pad prism was used to calculate IC_50_ values. Correlation between IC_50_ values of different assays with total flavonoid and total phenolic content was calculated by Pearson^’^s correlation coefficient with GarphPad Prism software version 5. The significance level was P < 0.05.

## Results

### Extraction yield

The *A. gracilis* crude methanlic extract yield was 17% of dry powder, while fractions AGHE, AGEE, AGBE and AGAE yield was 30, 12, 22 and 36%, respectively of dry crude methanol extract used.

### Total phenolics and flavonoids contents

Phenolics and flavonoids contents profile of *A. gracilis* extract and different fractions were estimated (Table 1). Total phenolic contents of *A. gracilis* samples were determined from the standard calibration curve (R^2^ = 0.96) of gallic acid. Crude methanolic extract (AGME) expressed (195 ± 5 mg GAE/g dry sample). It varied from 21 ± 1.5 to 615 ± 13 mg GAE/g dry sample after fractionation of methanolic extract in fractions. The maximum quantity of total phenolic contents (TPC) was observed in AGEE (615 ± 13 mg GAE/g dry sample) followed by AGBE (311 ± 10 mg GAE/g dry sample). AGAE showed 109 ± 6.1 mg GAE/g dry sample while lowest quantity was concentrated in AGHE (21 ± 1.5 mg GAE/g dry sample). All samples showed significantly (P < 0.05) different quantity of total phenolics.

**Table 1:** **Total phenolic and total flavonoid contents of** ***Asparagus gracilis*** **crude methanol extract and its derived fractions**

Sample	TFC (mg rutin equivalent/g dry sample)	TPC (mg gallic acid equivalent/g dry sample)
AGME	204.5 ± 5.0^b^	195.0 ± 5.0^c^
AGHE	9.7 ± 1.5^e^	21.0 ± 1.5^e^
AGEE	240.6 ± 6.1^a^	615.0 ± 13^a^
AGBE	151.7 ± 7.6^c^	311.0 ± 10^b^
AGAE	102.5 ± 5.1^d^	109.0 ± 6.1^d^

Values are expressed as mean ± SD (N = 3), Means with superscripts^a-e^ with different letters in the rows are significantly (P < 0.05) different from each other. TPC, Total Phenolic Content; TFC, Total Flavonoid Content.

The total flavonoid content of *A. gracilis* extract and fractions was estimated from a standard calibration curve of rutin (R^2^ = 0.93). Methanolic extract (AGME) showed 204.5 ± 5 mg RE/g dry sample flavonoids content. In fractions of AGME, maximum quantity was estimated in AGEE (240.6 ± 6.1 mg RE/g dry sample) followed in descending order of AGBE (151.7 ± 7.6 mg RE/g dry sample) > AGAE (102.5 ± 5.1 mg RE/g dry sample) > AGHE (9.667 ± 1.5 mg RE/g dry sample).

### HPLC analysis

HPLC-DAD was used to identify and quantify flavonoids and phenolics in crude methanolic extract. AGME by HPLC-DAD showed several chromatographic peaks but only five peaks were identified by comparing the retention time of peaks with known compounds used as standards. Compounds identified at different retention time were caffeic acid; 11.303, catechin; 12.723, rutin; 16.434, myricetin; 18.684 and quercetin; 20.537. Quantitative analysis of peaks identified resulted in catechin (4.04 ± 0.02 μg/mg sample), caffeic acid (0.89 ± 0.003 μg/mg sample), rutin (24.58 ± 0.1 μg/mg sample), myricetin (1.13 ± 0.07 μg/mg sample) and quercetin (14.91 ± 0.09 μg/mg sample) as given in Table 2.

**Table 2:** **Quantification of different compounds in methanol extract(AGME) of** ***Asparagus gracilis***

Phenolics/Flavonoid	Retention time(min)	Quantity(μg/mg AGME)
Caffeic acid	11.303	0.89 ± 0.01
Catechin	12.723	4.04 ± 0.02
Rutin	16.434	24.58 ± 0.10
Myricetin	18.684	1.13 ± 0.07
Quercetin	20.537	14.91 ± 0.09

Values are expressed as mean ± SD (N = 3).

### Antioxidant capacity

#### DPPH radical scavenging activity

The IC_50_ values of DPPH radical scavenging activity are given in Table 3. Best IC_50_ was shown by AGEE (76 ± 1.1 μg/ml) followed by AGAE (133 ± 2.5 μg/ml), while highest IC_50_ value was shown by AGHE. For IC_50,_ descending order of AGEE < AGAE < AGBE < AGME < AGHE was observed. Extract and its derived fractions showed good correlation with TPC (R^2^ = 0.62) as well as TFC (R^2^ = 0.64) but a non significant (P > 0.05) difference was observed, as shown in Table 4.

**Table 3:** **IC**_**50**_ **values of different antioxidant activities of extract and derived fractions of** ***Asparagus gracilis***

Activity	IC _50_(μg/ml)					
	AGME	AGHE	AGEE	AGBE	AGAE	Standard
DPPH scavenging activity	188 ± 2.1^b^	319 ± 4.1^a^	76 ± 1.1^e^	141 ± 1.2^c^	133 ± 2.5^d^	25.09 ± 0.8^f^
Hydrogen peroxide scavenging activity	213 ± 3.2^b^	452 ± 4.8^a^	113.5 ± 1.5^e^	182.3 ± 1.9^d^	201 ± 2.1^c^	99 ± 1.1^f^
Hydroxyl radical scavenging activity	201 ± 1.7^c^	348.5 ± 2.3^a^	98.3 ± 0.9^e^	127.4 ± 1.1^d^	228 ± 1.3^b^	32 ± 0.7^f^
ABTS scavenging activity	302 ± 3.1^b^	428.2 ± 4.6^a^	69.5 ± 1.5^e^	160 ± 1.4^d^	217 ± 2.1^c^	55 ± 1.4^f^
Anti Lipid per oxidation activity	237 ± 2.4^b^	312 ± 2.3^a^	77.3 ± 1.5^e^	140 ± 1.3^d^	176 ± 1.4^c^	31 ± 0.6^f^
β- carotene bleaching scavenging activity	187 ± 2.3^b^	428 ± 5.6^a^	115 ± 1^e^	128 ± 1.2^d^	225 ± 2.4^c^	35 ± 1.8^f^
Superoxide radical scavenging activity	172 ± 2.7^b^	435 ± 6.1^a^	73 ± 4.2^e^	107 ± 1.4^d^	147 ± 3.2^c^	41 ± 0.6^f^
NO^-^ radical scavenging activity	200 ± 3.7^b^	195 ± 2.4^b^	160 ± 2.9^c^	600 ± 4.6^a^	210 ± 2.1^b^	40.9 ± 2.3^d^

Values are expressed as mean ± SD (N = 3), Means with superscripts^a-f^ with different letters in the column are significantly (P < 0.05) different from each other.

**Table 4:** **Correlation of IC**_**50**_ **values of different antioxidant activities with total phenolic content and total flavonoid content**

Activity	Correlation R ^2^	
	TFC	TPC
DPPH scavenging activity	0.64	0.62
Hydrogen peroxide scavenging activity	0.77^a^	0.60
Hydroxyl radical scavenging activity	0.75^a^	0.78^a^
ABTS scavenging activity	0.56	0.77^a^
Anti Lipid per oxidation activity	0.54	0.73^a^
β - carotene bleaching scavenging activity	0.78^a^	0.61
Superoxide radical scavenging activity	0.74^a^	0.54
NO radical scavenging activity	0.71^a^	0.39

Values are expressed as mean. Means with superscripts^a^ have significant (P < 0.05) correlation with antioxidant activity mentioned in first colom. TPC, Total phenolic content; TFC, Total flavonoid content.

#### Hydrogen peroxide radical

The scavenging activity of various *A. gracilis* samples was observed to be concentration dependent. AGEE showed lowest IC_50_ of 113.5 ± 1.5 μg/ml against hydrogen peroxide radical, followed by AGBE < AGAE < AGME < AGHE (Table 3). The IC_50_ values of extract and all fractions were significantly higher than standard ascorbic acid. A significant correlation was observed between hydrogen peroxide scavenging activity and TFC (P < 0.05, R^2^ = 0.77, Table 4) while a non significant with TPC (P > 0.05, R^2^ = 0.6).

#### Hydroxyl radical scavenging activity

The *A. gracilis* samples scavenged •OH radicals and prevented 2-deoxyribose breakdown in this assay. A concentration dependent pattern was observed for hydroxyl radical scavenging activity. Lowest IC_50_ value was shown by AGEE and AGBE fractions (98.3 ± 0.9 and 127.4 ± 1.1 μg/ml respectively), while the highest was observed for AGHE (348.5 ± 2.3 μg/ml). Overall, AGEE < AGBE < AGME < AGAE < AGHE order was observed (Table 3). A significant (P < 0.05) correlation was observed with TFC as well as TPC (Table 4).

#### ABTS radical scavenging activity

The ABTS radical scavenging activity of *A. gracilis* samples was evaluated by using 2,2 azobis-(3-ethylbenzothiozoline-6-sulphonic acid). The best activity against ABTS radical was shown by AGEE (69.5 ± 1.5 μg/ml) while the lowest by AGHE (428.2 ± 4.6 μg/ml), as shown in Table 3. A significant correlation (P < 0.05, R^2^ = 0.77, Table 4) was observed between ABTS radical scavenging activity IC_50_ values with TPC, while a non significant correlation was observed with TFC (P > 0.05).

#### Anti lipid peroxidation potential

The IC_50_ values of anti-lipid per oxidation activity of *A. gracilis* extract and its derived various fractions are given in Table 3. Highest antilipid per oxidation activity was observed by AGEE and lowest by AGHE with IC_50_ values of 77.3 ± 1.5 μg/ml and 312 ± 2.3 μg/ml, respectively.

#### Beta carotene scavenging activity

Solvent polarity based scavenging activity was shown by *A. gracilis* extract and its various fractions. The lowest IC_50_ (115 ± 1 μg/ml) was recorded for AGEE, while highest (428 ± 5.6 μg/ml) for AGHE (Table 3). This study shows that AGEE has notable activity in minimizing the loss of beta carotene during the coupled oxidation of linoleic acid and beta carotene in the emulsified aqueous system. Beta carotene assay showed a significant correlation with TFC.

#### Superoxide radical scavenging activity

Extract as well as its all fractions recorded good superoxide radical scavenging activity. The highest activity was observed from AGEE and lowest from AGHE. Descending order of AGEE < AGBE < AGAE < AGME < AGHE for IC_50_ was estimated from superoxide radical scavenging activity assay (Table 3). The total flavonoid contents showed a significant (P < 0.05, Table 4) correlation with superoxide radical scavenging activity.

#### Nitric oxide assay

In the present study, the lowest IC_50_ value was exhibited by AGEE (160 ± 2.9 μg/ml) while highest by AGBE (600 ± 4.6 μg/ml). Descending order of AGEE < AGHE < AGME < AGAE was observed as displayed in Table 3.

#### Reducing power

Good reducing power was shown by AGEE, comparable to standard gallic acid, followed by AGBE > AGME > AGAE > AGHE.

#### Total antioxidant assay

Maximum total antioxidant activity was shown by AGEE and followed by AGBE > AGME > AGAE > AGHE at the highest dose of 200 μg/ml.

#### DNA protection study

**Figure 1:**
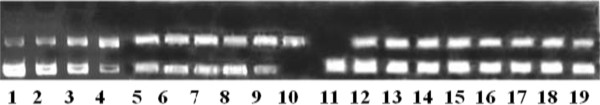
**DNA protection assay of crude methanol extract and various fractions of** ***Asparagus gracilis*** **{l: Control, 2: 2 mM FeSO**_**4**_ **, 3: 30% H**_**2**_ **O**_**2**_ **, 4: 2 mM FeSO**_**4**_ **+ 30% H**_**2**_ **O**_**2**_ **,5: AGME (100 μg/ml), 6: AGME (50 μg/ml), 7: AGME (25 μg/ml), 8: AGBE (100 μg/ml), 9:AGBE (50 μg/ml), 10: AGBE (25 μg/ml), 11: AGEE (100 μg/ml), 12: AGEE (50 μg/ml), 13: AGEE (25 μg/ml), 14: AGHE (100 μg/ml), 15: AGHE (50 μg/ml), 16: AGHE (25 μg/ml),17:AGAE (100 μg/ml),18:AGAE (50 μg/ml),19:AGAE (25 μg/ml)}.** AGME; *Asparagus gracilis* crude methanol extract. AGEE; *Asparagus gracilis* ethyl acetate fraction. AGBE; *Asparagus gracilis n*-butanol fraction. AGAE; *Asparagus gracilis* aqueous fraction. AGHE;*Asparagus gracilis n*-hexane fraction.

FeSO_4_ and H_2_O_2_ treatment individually showed two bands but the lower band was more concentrated, indicating a minor damage of DNA. However, FeSO_4_ + H_2_O_2_ expressed a damaged form of plasmid DNA. Crude methanol extract and its derived fractions showed low to high degree of protection of plasmid DNA against Fenton reaction induced degradation. Best DNA protection was displayed by AGEE fraction. AGME, AGAE, AGHE and AGBE showed low level of protection. The results of this experiment suggest that AGEE has some potent antioxidant component; scavenging hydroxyl radical produced in the Fenton reaction and protects DNA from damage (Figure 1).

### Antiurease activity

**Figure 2:**
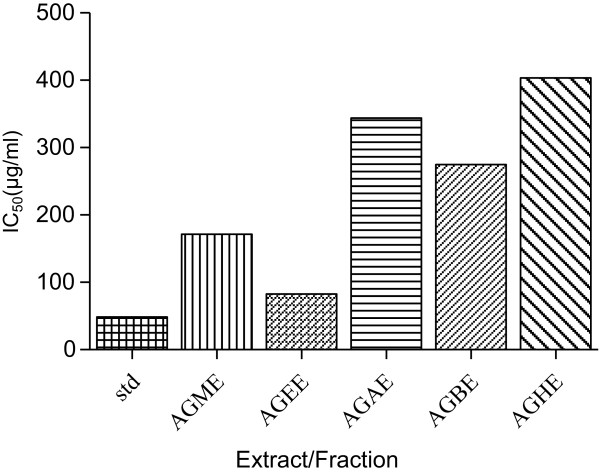
**IC**_**50**_ **values of anturease activity of** ***Asparagus gracilis*** **crude methanol extract and its derived various fractions.** AGME; *Asparagus gracilis* crude methanol extract. AGEE; *Asparagus gracilis* ethyl acetate fraction. AGBE; *Asparagus gracilis n*-butanol fraction. AGAE; *Asparagus gracilis* aqueous fraction. AGHE; *Asparagus gracilis n*-hexane fraction.

To evaluate this property, antiurease activity of *A. gracilis* extract and its derived fraction was performed by using jack bean urease enzyme. Crude methanolic extract showed IC_50_ value of 171.6 μg/ml. AGEE proved most active among fractions against urease and showed IC_50_ value of 82.86 μg/ml followed by AGBE with IC_50_ of 274.6 μg/ml. AGAE and AGHE were less active showing IC_50_ value of 344 μg/ml and 403.6 μg/ml, respectively (Figure 2). Analysis showed significant correlation with flavonoids (R^2^ = 0.93, P < 0.004) and phenolics (R^2^ = 0.75, P < 0.02).

## Discussion

Extract and fraction yield vary with the nature of the solvent used. In the present study, aqueous fraction gave maximum yield and ethyl acetate yield was minimum among solvents used. Shah et al., [21] observed different yield of various fractions working on *Sida cordata*.

Phenolic compounds constitute one of the major classes of compounds which act as natural antioxidants and this property make it a potential agent in oxidative problems. Therefore it was reasonable to concentrate it in a fraction by using organic solvents of different nature i.e. *n*-Hexane, Ethyl acetate and *n*-Butanol. Phenolics and flavonoids contents of *A. gracilis* extract and different fractions were estimated. All fractions expressed significantly different quantity of total phenolics. Ethyl acetate proved best solvent to elucidate flavonoids and phenolics from the methanolic extract. Statistically, extract and its derived fractions showed significantly different quantity of total phenolic and flavonoid contents. Almost similar changes in total phenolic content and total flavonoid content were reported by Khan et al. [22] and Saeed et al. [11] in fractions derived from crude methanol extracts. HPLC-DAD analysis of AGME expressed important phenolics i.e. caffeic acid, catechin, rutin, myricetin and quercetin.

Antioxidant activity is a complex process usually occurring through several mechanisms. In light of the differences among the wide number of test systems available, the results of a single assay can give only a reductive suggestion of the antioxidant properties of extracts towards food matrices and must be interpreted with some caution. Moreover, the chemical complexity of extract/fraction, often a mixture of dozens of compounds with different functional groups, polarity and chemical behavior, could lead to diverse results, depending on the tests employed. Therefore, an approach with multiple assays in screening work is highly advisable due to its complexity and followed in the present study.

Radical scavenging activities are very important to prevent the harmful effects of free radicals which can lead to certain health hazards and food spoilage in food industry. Excessive free radical generation promotes lipid oxidation in foods leading to low quality food and consumer's refusal. The DPPH molecule, which contains a stable free radical, has been widely used to evaluate the radical scavenging ability of antioxidants. In the DPPH assay, the antioxidants are able to reduce the stable radical DPPH to the yellow colored diphenylpicrylhydrazine. The method is based on the reduction of alcoholic DPPH solution in the presence of a hydrogen donating antioxidant due to the formation of the non-radical form DPPH–H. With this method it is possible to determine the antiradical power of an antioxidant activity by measuring of a decrease in the absorbance of DPPH at 517 nm, resulting in a color change from purple to yellow, the absorbance decreased when DPPH is scavenged by an antioxidant through donation of hydrogen to form a stable DPPH molecule. In the radical form, this molecule has an absorbance at 517 nm which disappear after acceptance of an electron or hydrogen radical from an antioxidant compound to become a stable diamagnetic molecule [23]. All the *A. gracils* samples showed high IC_50_ values than ascorbic acid. Though DPPH radical scavenging activity is significantly lower (P < 0.05) than the standard ascorbic acid in comparison (Table 1) but AGEE, AGAE, AGBE as well as AGME showed good antioxidant activity and may be attributed to the presence of good quantity of TPC and TFC in these samples. Results obtained in this study suggest that DPPH scavenging activity can be enhanced by the partition of crude methanol extract with ethyl acetate organic solvent. Similar results were observed [24] in their study on *Fagonia olivieri* whole plant. However, not in agreement with the study of Bokhari et al. [25], where they observed maximum DPPH scavenging activity in aqueous fraction of *Gallium aparine*.

Hydrogen peroxide is the reactive oxygen metabolite causing damage to the cell at very low concentration of 10 μM. It is produced as a result of dismutation of superoxide radicals or directly or indirectly by some enzymes. Free solubility in aqueous makes it freely movable across biological membrane. Deleterious effects include degradation of heme protein, inactivation of enzymes and oxidation of DNA, lipids, -SH groups, and keto acid [26]. It reacts with Fe^2+^ and possibly Cu^2+^ ions to form hydroxyl radicals, which induce many toxic effects [27]. In the present study, IC_50_ values of extract and fractions were observed significantly higher than the standard ascorbic acid but a significant correlation was observed with total flavonoid contents. This difference may be due to the stoichiometry of reactants of both the classes resulting in high and low output [28]. Ethyl acetate fraction exhibited lowest IC_50_ among the different fractions. These results are in agreement to the previous study [24], reporting ethyl acetate fraction for high hydrogen peroxide scavenging activity.

Hydroxyl is short lived, toxic free radical having affinity to other molecules. It is a potent oxidizing agent and reacts with a very high rate with most inorganic and organic molecules including lipids, protein, amino acids and deoxyribonucleic acids, leading to cancer, mutagenesis and cytotoxicity. It reacts with other molecules by hydrogen abstraction, addition and electron transfer [26]. The hydroxyl radical is generated in chemical reactions in the human body. Superoxide dismutase converts the superoxide oxide into hydrogen peroxide which is converted to a highly reactive hydroxyl radical. In the present experiment, the evidence of •OH scavenging activity by *A. gracilis* extract and its fractions were obtained through the deoxyribose system. Hydrogen peroxide is reacted with ferrous and hydroxyl radical was produced which reacted with deoxyribose. The reaction was stopped by thiobarbituric acid. A red color developed after reaction of hydroxyl radical with deoxyribose. Scavenging activity of hydroxyl radical is directly proportional to the antioxidant activity of the extract/fraction, observed by the low intensity of red color [29]. Results obtained in this study are in agreement to to the study of Shah et al. [24]. The strong antioxidant activity of AGEE and AGBE can be utilized as a source of natural antioxidant in oxidative stress for minimizing the detrimental effects of hydroxyl radical in the body.

ABTS is routinely used methodology for estimation of antioxidant potential of plants and pure compounds. ABTS^+^ is a blue chromophore produced by the reaction between ABTS and potassium per sulfate. The addition of the antioxidants to this pre-formed radical cation reduces it to ABTS in a concentration dependent manner. This method of decolorization assay of antioxidant activity is applicable to both hydrophilic and lipophilic antioxidants including carotenoids, flavonoids, hydroxycinnamates and plasma antioxidants [29]. Ethyl acetate fraction showed lowest IC_50_ value in scavenging of ABTS radical. Significant correlation was observed with total phenolic content, showing activity might be due to the presence of phenolics. ABTS radical scavenging activity of hydrogen donating antioxidant was observed by Sahreen et al. [30] in their study.

Peroxidation of lipids, predominantly polyunsaturated fatty acid residues of phospholipids and cholesterol esters is a route of striking implications. It shortens the shelf-life of food and drugs, causes fragmentation of DNA, damages cellular membranes and promotes the genesis of many human diseases, metabolic malfunctioning and aging. Therefore, researchers are committed to search for a potent antioxidant. Egg yolk lipids on reaction with ferrous sulphate undergo rapid non enzymatic per oxidation. Highest antilipid peroxidation activity was expressed by ethyl acetae fraction, but in opposite to Ahmad et al. [13] reports on *Dicliptera roxburghiana*. They noted highest IC_50_ value for crude methanol extract in comparison to ethyl acetate fraction. IC_50_ values are comparable with standard but significantly different. Significant correlation was observed with total phenolic content.

The antioxidant potential of *A. gracilis* extract and different fraction was determined by a beta carotene bleaching method based on the oxidation of linoleic acid. Linoleic acid hydroperoxides react with beta carotene molecule resulting in the rapid disappearance of color. The presence of the antioxidant extract can obstruct the extent of beta carotene by acting on linoleate free radicals and other free radicals formed in the system. Therefore, the absorbance rapidly decrease in samples without antioxidants, whereas in the presence of an antioxidant, they maintaine their absorbance and color for a longer period [31]. In the present study ethyl acetate illustrated lowest concentration in maintaining the color of beta carotene, so expressing high antioxidant activity.

Oxidation is an important phenomenon of life, but apart from so many crucial processes of life, during normal metabolism of oxygen, various free as well as superoxide’s are continuously produced. It is considered a weak oxidant, but gives rise to toxic and powerful oxidant such as hydroxyl radical and singlet oxygen resulting in many diseases [11].

Compared with other oxygen radicals, superoxide anion has a longer lifetime, can move a long distance, and thus can be dangerous for the affected or associated systems. Therefore, it is very important to study the scavenging of superoxide anion. Superoxide anion derived from dissolved oxygen by PMS–NADH coupling reaction reduces NBT in this system. In this method, superoxide anion reduces the yellow dye (NBT^2+^) to produce the blue formazan which is measured spectrophotometrically at 560 nm. Antioxidants are able to inhibit the blue NBT formation. The decrease of absorbance at 560 nm with antioxidants indicates the consumption of superoxide anion in the reaction mixture [29]. Concentration dependent superoxide radical scavenging activity was shown by extract and fractions of *A. gracilis*. A significant (P < 0.05) correlation of superoxide radical scavenging activity was observed with total flavonoid contents. Highest activity was exhibited by ethyl aceate fraction. The results observed for the superoxide radical scavenging activity are in contrary to other studies where they noted lowest IC_50_ of the aquesous fraction [32].

Nitric oxide is a very unstable species under aerobic condition. It reacts with O_2_ to produce its stable product nitrate and nitrite through intermediates NO_2_, N_2_O_4_ and N_3_O_4_. It is estimated by using Griess reagent. In the presence of a scavenging test compound, the amount of nitrous acid will decrease and can be measured at 546 nm. Nitric oxide is reported to play key role in various inflammatory processes. Its chronic expression leads to various diseases i.e. various carcinomas and inflammatory conditions including arthritis, juvenile diabetes, multiple sclerosis and ulcerative colitis [27]. Toxic and highly reactive peroxynitrite anions (ONOO-) are formed when NO radical reacts with superoxide radical [28]. Sodium nitroprusside generates nitric oxide which reacts with oxygen to yield nitrite. The antioxidants inhibit nitrite formation by directly competing with oxygen in the reaction with nitric oxide. The present study proved that the extract/fractions of *A. gracilis* studied have good nitric oxide scavenging activity.

Reducing power of the plant was determined by using the potassium ferricynide reduction method. In reducing power assay, the oxidation form of iron (Fe^+3^) in ferric chloride is converted to ferrous (Fe^+2^) by antioxidant compounds resulting in conversion of yellow color of the test solution to green. The intensity of green color formation is directly proportional to the reducing power of the sample. High reducing power samples show high absorption at 695 nm in spectrophotometer. Literature attributes reducing power to the hydrogen donating ability of antioxidants to the free radicals [33]. Marked reducing power was illustarted ethyl acetate fraction in comparison to other fractions. The phytochemicals present in the AGEE caused reduction of Fe/Ferricynaide complex ferrous ion and thus showed reducing power. Present results are not in accord to Ahmad et al. [13], where highest activity was recorded in crude methanol extract. Now it is an established phenomenon that reducing power is linked with antioxidant potential and it correlates with phenolic constituents in several vegetables/foods. The reducing power of a compound may serve as an important marker of its possible antioxidant activity. However, the activity of antioxidants have been ascribed to various mechanisms such as prevention of chain initiation, decomposition of peroxides, reducing capacity and radical scavenging [34].

Phosphomolybdenum assay principal follows the chemistry of conversion of Mo(VI) to Mo(V) by compound/extract having antioxidant property and resulting in formation of green phosphate/Mo(V) compound with maximum absorption at 695 nm at acidic pH. Results od total antioxidant activity are in agreement to Shah et al. [35] reports on *Jurinea dolomiaea* roots. Electron and hydrogen transfer from antioxidant compound/extract to Mo(VI) complex take place in the phosphomolybdenum assay methods. The transfers of electrons or hydrogen depend on the structure of the antioxidant and redox potentials in the assays [36].

Reactive oxygen species impose damages on cellular structures and functional machinery, including DNA, even under normal physiological circumstances. In the present study, hydroxyl radicals were generated by Fenton reaction, attach to DNA, progress to base alteration, deoxyribose fragmentation and strand breakage. Besides this, oxidation of lipids induced by •OH and other ROS can produce unsaturated aldehydes and malondialdehyde, that can react with DNA and leads to mutagenic adducts [37]. In pBR322 DNA gel electrophoretic pattern, the band with faster movement represent the native form of super coiled plasmid circular DNA and the band moving slower correspond to the open circular form (Figure 1). The data of present study suggest that ethyl aceate fraction of *A. gracilis* has some potent antioxidant component; scavenging hydroxyl radical produced in the Fenton reaction and protects DNA from damage. Similar results were observed by Shah et al. [35] in their study but with fractions other than ethyl acetate. Number of exogenous agents including metal ions is responsible for oxidative damage to DNA i.e. intra-linked bases, depurination and nicked strands [38]. In Fenton reaction, ferrous ions reduce H_2_O_2_ and generate free •OH radicals [39]. Malondialdehydes are formed by the attachment of DNA deoxyribose to these free •OH radicals [40]. According to Spotheim et al. [41], the disruption of plasmid DNA is the consequence of breakage in the phosphodiester bonds, forming an open and linear structured DNA. Zhao et al. [42] reported verbacoside as a ligand of free iron ions, preventing hydroxyl radicals mediated pBR322 damage. Flavonoids were discovered to express the same plasmid protective ability [43]. According to Walia et al. [44] polyphenolic possess scavenging activity; this protective potential can be the result of hydrogen donating capability. Thus, they can be involved in transformation of hydrogen peroxide to water. Polyphenolic can also irreversibly bind to the active sites of Fe2+, causing it to attain an inert state.

Many naturally existing antioxidant compounds in different plants have a multi potent effect. A crucial role is played by these multi potent antioxidants against diseases. Free radicals are involved in pathogenesis and mostly more than one pathogenic factor contributes to a disease. Thus, discovery of multi effective compounds instead of single targeting molecules is essential [45]. In the present study, ethyl acetate fraction of *A. gracilis* proved most active among fractions against urease and showed lowest IC_50_ value. Similar results were reported by Lateef et al. [46] in their study on roots of *Glycyrrhiza glabra*, where ethyl acetate fraction showed maximum inhibitory activity against urease. Approximately similar IC_50_ range was shown by *Sambucus ebulus* and *Rheum ribes* plants in a study performed by Nabati et al. [2]. Similar, patterns of variation in antioxidant activity of different fractions were observed, which give rise to a possibility of multipotent activity of antioxidant compounds in the fractions.

## Conclusion

Results obtained in the present study show that *A. gracilis* has strong antioxidant and antiurease activity. Further study is required to isolate compounds in pure responsible for the activity, may prove leads for enhanced antioxidant potential agent in food industry as well as human health.
